# Optimization of Walk Score Based on Street Greening—A Case Study of Zhongshan Road in Qingdao

**DOI:** 10.3390/ijerph18031277

**Published:** 2021-01-31

**Authors:** Ye Sun, Wei Lu, Peijin Sun

**Affiliations:** School of Architecture and Arts, Dalian University of Technology, Dalian 116081, China; jianyisy2019@mail.dlut.edu.cn (Y.S.); sunpeijin@dlut.edu.cn (P.S.)

**Keywords:** walking index, daily life facilities, green vision rate, geographic information system (GIS)

## Abstract

Enhancing the walkability of urban streets is an effective means to improve public health, alleviate traffic congestion, and enhance the living environment. In China, the government has actively encouraged green travel and promoted improvements in the walk system. The walkability of the built environment is affected by many factors. In addition to the configuration of daily life facilities, street greening can have significant effects on walkability. To explore the rationality of street life facilities and understand the impact of the natural attributes of the block space (street-level greening) on the quality of the walking environment, we evaluated the walkability of Zhongshan Road in Qingdao, China and optimized the algorithm of the walk score. In this study, we selected residential areas as the starting point and modified the weight coefficients for facilities to evaluate the walkability of streets. Traditional research methods were combined with street view image capture, and the rate of the attenuation factor was used for the new optimization algorithm. We discussed the rationality of street life facilities and increased the green vision rate using a correction index. By comparing changes in walkability before and after joining, we analyzed the necessity of including new indicators. The results show that the average walking index of Zhongshan Road is 79.74, and the overall performance is good, showing a high trend in the west and a low trend in the east, and a high trend in the south and a low trend in the north. According to the general walking index, western stations and southern coastal areas have higher scores, and living facilities are well equipped; old northern and eastern residential areas have lower scores. Among them, the average weight of bookstores is 0.74, and the average weight of parks is 0.69. To meet residents’ needs for daily leisure activities, adding bookstores or similar facilities in community parks would be necessary to improve daily facilities and services. The average green viewing rate of Zhongshan Road is 20.48%, which is lower than the best visual perception value of 25.00%. Comparing the walking index changes before and after adding the green viewing rate, the high-scoring area shifted from the west to the south, and the west walking index has the most significant decline. Street greening has a certain impact on the quality of the walking environment. The results and conclusions of this study can be used as a reference in developing street walkability indicators and further improving the evaluation system.

## 1. Introduction

In the World Health Organization’s global public health report released in 2017, 27.5% of the world’s population suffer from insufficient exercise [1]. Walking is a relatively relaxed and unrestricted sports activity [2], and most people can complete it in the streets and open spaces within urban communities. The Chinese government issued the “Green Travel Action Plan” in 2019, giving walking the right to allocate and balance road traffic resources to build a safe, continuous, and comfortable urban pedestrian road system. Therefore, it is particularly important for urban planners to understand the construction of walkable places. Existing research has shown that factors at different levels, from the built environment, street accessibility, and rationality of living facilities, can affect people’s walking behavior [3,4,5]. Studies have shown that the land use structure and street connectivity affect walkability at the macro-level [6,7]. At the micro-scale, the walk score is an effective and reliable method to quantify walkability [8,9,10,11,12]. It can reflect the rationality of daily facility configuration within a certain walking range.

Many factors in the built environment affect a community’s walkability and the population’s walking behavior. Examples include the microscopic walkability determined by pedestrian perception, the attractiveness of the street scene, and the condition of the sidewalk in the block [13,14,15]. Numerous studies have evaluated walkability in different parts of the world, using GIS [16,17,18], Lawton’s ecological model of aging and LTPA scale (leisure-time physical activity) [19,20], internet-delivered pedometer [21], and wearable sensors [22]. Other tools and methods have also been used to explore the correlation between measurement values [22]. While many of these studies have shown that the walking index can be used as an effective indicator to measure street walkability, the algorithm used in calculating the walking index does not involve natural walking obstacle factors, such as water systems, topography, mountains, and greenery. Later, scholars found that by adding indicators, such as the proportion of open space, green coverage, and employment density, the walking index would be more representative [23,24,25]. Traditional research methods and social observation require integrating time and resources [26], which cannot be satisfied in many large-scale research projects. Street view images have become a new trend for evaluating the micro-walkability of large areas [27,28]. Existing studies have shown that the attractiveness of micro-scale street views can effectively promote pedestrian walking [13,15,29]. Using Google Street View (GSV) to evaluate micro walkability, Ewing et al. found a positive correlation between street view characteristics and pedestrian activities [14,27,28].

Previous research has mostly focused on the social attributes of street space. Many have used street view image recognition and machine learning to measure walkability on a large scale for different groups of people, often ignoring the impact of natural attributes. To supplement the influence of natural factors on walkability in the urban natural environment, this study explores the impact of street greening on walking [30]. We combined traditional research methods with street view acquisition and added the green viewing rate index in street view images to optimize the traditional algorithm. The main purpose of this study is to measure the walkability of Zhongshan Road through the walk score and evaluate the rationality of living facility allocation at the walking level. We explored the influence of the street’s natural factors on walkability and compared the difference between the measurement results before and after incorporating the new index. We provided a more objective evaluation for street walkability, which better reflects the walkability of the space. Research is important to enhance walkability, improve the layout of living facilities, and optimize the walking index algorithm. It also provides quantifiable guidance and suggestions for urban planning and design.

## 2. Methods

### 2.1. Study Design and Data Collection

For 2019, in the “Evaluation of Pedestrian Friendliness in Chinese Cities” [31,32] report, the average value of the pedestrian index for Qingdao’s urban streets in Shandong, China was 76.00 points. The overall performance was good, but there was a huge gap between indicators, including street greening and first-tier cities. According to the “Large Scale Measurement of the Pedestrian Index of China’s Main Streets” and using data from Jihai [33], Qingdao’s South District pedestrian index scored 80.80, which indicates that it is very suitable for walking. 

**Figure 1:**
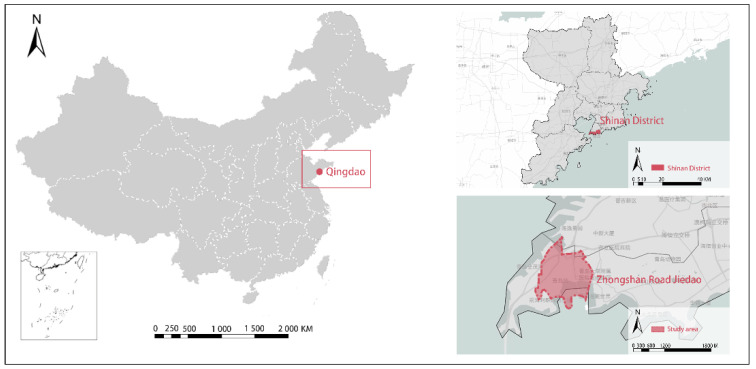
Location of the study area.

For this study, the research area is Zhongshan Road in Shinan District, Qingdao (Figure 1). The street has a high degree of construction and a large number of tourist attractions. Numerous buildings with distinct historical features are found in the area, which can effectively encourage citizens and tourists to choose walking [34]. The street covers an area of 1.71 km^2^ with a current permanent population of 22,573 people (Table 1).

In calculating the universal walking index, this study makes use of residential quarters as the travel point and sets the living range for 5, 10, and 15 min. Regional adjustments were made to the facility weight coefficient, and the intersection density was obtained after determining the basic walking index. The block length and the green viewing rate index were revised, and the overall street walkability and the facility configuration were then evaluated. The road network data used in this study were obtained from the Open Street Map website, and the vector road network information was edited and processed in ArcGIS. The urban daily life facility data POI (Point of Interest, A POI can be a house, a shop, a mailbox, a bus stop, etc) was obtained from the network open-source data, which were then corrected and classified on a third-party website. The street greening data were acquired from Baidu street view images, and the green viewing rate was determined using the maoyan quadrant program.

### 2.2. Procedure

The road network topology was processed and expressed as a single-line network to allow the marking of the street view images. The equivalent time-circle road network was constructed in terms of 5 min living circles, 10 min living circles, and 15 min living circles [35] and standard pace to form different walking distances. The 1031 POI information points obtained were classified, and the weight coefficients were adjusted according to regional habits. The single-point walking index was calculated using the weight classification table to determine the basic walking index (Table 2). After the intersection density and the block length attenuation were corrected, the general walking index was obtained using the Kriging interpolation method and was then used to analyze the street daily life facility configuration. The average green vision rate around the travel point was calculated and analyzed, and the optimized walking index could then be finally determined. 

### 2.3. Optimization of the Facility Weight of the Walk Score

In 2007, the United States proposed the walk score classification weight [8], which added relevant facilities and adjusted the weight values according to actual needs. For example, Zhongshan Road is located in the center of the city. The density of commercial service facilities is high, attracting much traffic, and the possibility of walking is great. There are trendy shops, including coffee shops (such as Starbucks) and milk tea shops (such as COCO, the name of a hot milk tea shop ), which are popular among young people and may promote walking in the area. For this study, a total of 6 categories and 13 subcategories were used, and the summary of the classification weight is provided in Table 2.

### 2.4. Distance Attenuation Function

**Figure 2:**
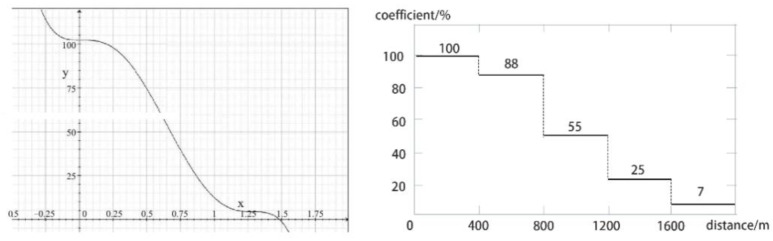
Distance attenuation curve and facility weight attenuation law.

The distance attenuation is based on the distance between the trip and the facility’s location to determine the percentage of weight. The parameter is usually given as a polynomial distance attenuation function [36,37,38], as shown in Figure 2. Studies have shown [8] that the best and most comfortable time for walking is 5 min, and the tolerance time is 20 min. Within the 5–20 min time range, the willingness to walk decreases as the walking time increases. For simplification purposes, the model uses a piecewise function. Using the centroid of the residential quarters in the study area as a starting point and a standard walking speed of 80m/min, GIS buffer analysis was used to delineate the 5 min, 10 min, and 15 min life circles [39] (Figure 2).

### 2.5. Green View Rate

Street greening is characterized by the greenness rate. Green vision rate refers to the proportion of green plants in the objects that people see. Compared with the greening rate and green space rate, the green vision rate can better reflect the quality of the public greening environment [40,41] (Table 3). Street view images were taken at a distance of 50 m. The average value from multiple locations for a given street determines the street’s green viewing rate. The standard deviation for the green viewing rate indicates the evenness (consistency) of the distribution. The larger the standard deviation, the more uneven the distribution. Due to the small size of the research area, the street green vision rate was processed using the cat’s eye quadrant program. The natural break (natural breakpoint classification method) was used for classification to obtain the average value and standard deviation of the entire area.

### 2.6. Correction of Basic Walking Index

According to Allan B. Jacobs’s “Great Streets” [42], people’s willingness to walk is affected by the street structure. The greater the density of the intersection, the stronger the walkability of the street. In contrast, as the dynamic visual richness of the street decreases, the walkability is reduced. In addition, the length of the block has a significant impact on the pedestrian environment. The attenuation rate of intersection density and block length is divided into five levels, with a maximum attenuation rate of 5% [3]. The green vision rate’s attenuation coefficient can be determined based on people’s highest visual perception of greenery (Table 4).

The total for the weights is equal to 15 points. To make the calculation results correspond to the walking index level, the value is scaled up to 100. The specific walking index calculation formula is as follows:

(1) $$\mathrm{Walk}\;\mathrm{Score}\;=\;\sum_{i}^{n}\left(wi\times Dt\right)\times \left(1-l\right)\times \left(1-u\right)\times \left(1-g\right)\times 100/15$$

where the Walk Score is the final score of the walking index, wi is the weight value of facility *i*, Dt is the corresponding coefficient of distance t in the attenuation function, *l* is the attenuation rate corresponding to street length, u is the attenuation rate corresponding to the number of intersections, and g is the attenuation rate corresponding to the green view rate. The walking index level [3] is shown in Table 5.

## 3. Results

### 3.1. Walking Coverage and Universal Walking Index

According to the walking coverage area, the residential area in the north is denser and can be reached within 15 min on foot, providing a distance basis for the configuration of living facilities. The south is composed mostly of tourist attractions, such as the trestle bridge and Guanhaishan Park, and the travel distance exceeds 15 min. 

**Figure 3:**
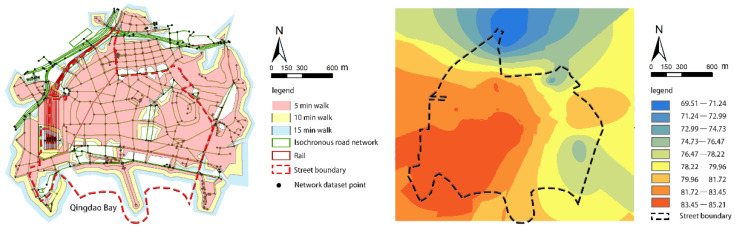
Walking coverage and general walking index.

The results of the general walking index analysis are shown in Figure 3. The overall street performance is good, showing a high trend in the west and a low trend in the east, and also a high trend in the south and a low trend in the north. The average walking index is 79.74, with the highest score being 85.21 and the lowest score being 69.51. The street has good walkability, and most places used for daily activities are within walking distance. Areas with high walking index are mainly concentrated near the western railway station, which has high density and high mobility. The high walking index in the southern coastal area is due to the high level of the street-built environment and the presence of various daily living facilities. The northern and eastern parts of the research area have old settlements and low public service levels. The facilities are mostly old, and the pedestrian index is generally low. The terrain on the east side of the street is undulating, which, to a certain extent, affects the walkability of the street.

### 3.2. Assessment of Living Facilities Configuration

**Figure 4:**
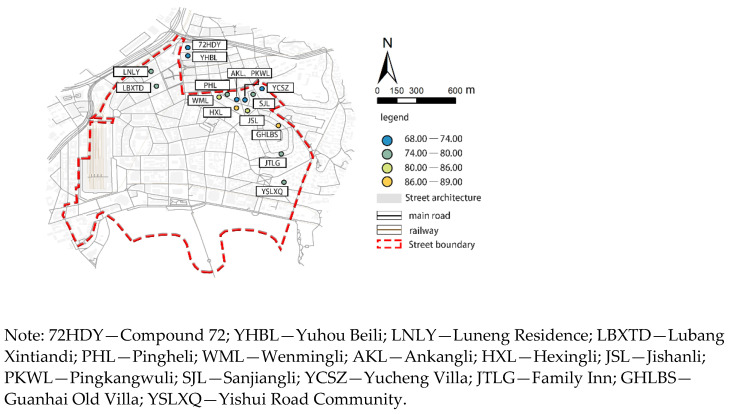
Community with low street walking index.

**Figure 5:**
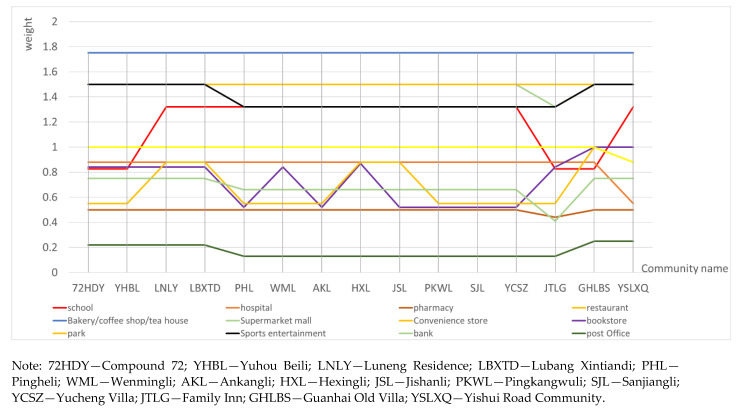
Weight statistics of living facilities in low-score communities.

The configuration of the daily life facilities in areas with low scores was further analyzed (Figure 4). The walking index scores for the various facilities at the selected travel point were calculated, and the impact of the configuration for the different types of living facilities at the travel point were evaluated (Figure 5, Table 6).

The results show that the average walking indexes for Ankangli, Pingkang Wuli, Sanjiangli, and Pinghewuli were less than 70.00. The walkability is average, and some facilities can be found outside the 15 min travel range. This phenomenon can be explained by a number of factors. First, the area is an old residential district with narrow roads and a shortage of parking spaces. Parking takes up huge portions of the streets and reduces walking space. Second, the public service facilities in the old residential area are of a few types and poor quality, mainly satisfying the basic daily life needs of the residents while neglecting their leisure and entertainment needs. Street greening is low, and some roads, such as Boshan Road and Weixian Road, have no greening amenities.

As shown in Table 6, public service facilities, such as hospitals, restaurants, bakeries, coffee shops, tea houses, shopping malls, convenience stores, and banks, scored higher in weight statistics. These types of facilities are more accessible and highly convenient. The average weight for parks is 0.69 (standard value 1.00) and 0.74 for bookstores (average value 1.00). The weights for these two types of facilities are low, and the fluctuation is large. It shows that the bookstores and parks as leisure facilities are inadequately equipped and have low accessibility, resulting in a low walking index. In addition, the fluctuations for educational, sports, and entertainment facilities indicate an uneven configuration problem. The lack of configuration is concentrated in the old residential areas, such as Pingheli, Ankangli, Jishanli, Pingkang Wuli, and Sanjiangli. The improvements in street walkability in later periods may be combined with urban renewal design to improve the level of facilities and life quality.

### 3.3. Street Greening and Integrated Evaluation

**Figure 6:**
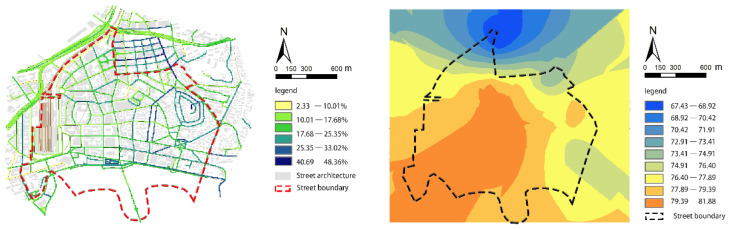
The pedestrian index of Zhongshan Road after superimposing the green viewing rate.

The overall green viewing rate for Zhongshan Road is low and unevenly distributed (Figure 6). The maximum value is 48.36%, and the minimum value is 2.33%. The average green viewing rate is 20.48%, which is lower than 25.00% (i.e., green eyesight rate that makes people feel comfortable). The standard deviation is 8.72, which suggests that the street greening is unevenly distributed. The extent is higher than 25.00% due to the natural landscape, the preferred coastal location, the high degree of street greening, and the high density of parks. The green viewing rates around the western railway station and the old residential areas in the north are low, with an average value of 15.17%. Wenmingli, Hexingli, Jishanli, and other residential clusters have no shrubs in the streets, and most trees are planted along one side, resulting in low green vision rates. Public spaces (e.g., streets and squares) around the railway station are relatively empty and lack green plants.

After adding the green vision rate, the walking index score drops (Figure 6). Although the walkability performance can still be characterized as good, the average value dropped 3.01. The highest score was 81.88, the lowest score was 67.43, and the average walk score was 76.73. After adding the new index, there is a significant difference in the pedestrian index within the street, and the areas with high regional walkability scores shifted from the west towards the south. These high-scoring areas were mainly concentrated around Zhongshan Road’s southern coastal roads, The green vision rate is between 25.35% and 40.69%, and the street greening environment is of high quality. The score decline is obvious around the western railway station. The green vision rate is between 2.33% and 17. 68%, and the walking index score is from 83.45–85.21 down to 77.89–79.39. Although the area has a high facility convenience score, the high-index construction density has compressed the street’s green spaces. Green plants in the activity square are scarce, considerably affecting the quality of the walking environment. The old residential districts in the north still had the worst walkability values in the region.

## 4. Discussion

### 4.1. Applicability for the Universal Walk Score

We chose a residential area that is more in line with the actual travel situation as the travel point. The public service facilities in the area can be reached on foot within 15 min. Compared with grid points, it is more accurate as a travel point. The average walking index obtained was 79.74. The street has good overall walkability, and most daily life activities and basic facilities are within acceptable walking distances. However, the difference between the highest (85.21) and lowest (69.50) walking scores in the region is 15.71. This disparity in walkability is caused mainly by the uneven distribution of living facilities, significant gaps between old and new infrastructure, and insufficient daily life facilities.

Our research shows that the universal walk score can provide a quick and effective scoring system for street walkability and that the POI information obtained with the help of open-source data can be applied for large-scale living facilities. The average walking index scores of the research results are 79.74 and 76.00 [22] (the average value of the Qingdao street walking index in the “China City Walkability Evaluation” issued by the Natural Resources Conservation Association) and 80.80 [23] compared with the average value of the pedestrian index in Shinan District of Qingdao, published by the Natural Resources Conservation Association and Tsinghua University of the “Evaluation of Walkability of Main Streets in China”, which proves its validity and reliability. However, note that the walk score cannot be used as an absolute standard for measuring walkability [8]. Instead, we recommend that the walk score be used as an assessment of daily life facilities. The allocation of service facilities and the indicators that quantify the overall walkability of neighborhoods can be used as a reference in urban planning and design.

### 4.2. The Impact of Daily Life Facility Configuration on Street Walkability

Using the results of the walkability index, we further analyzed the configuration of living facilities and found that different types of living facilities were found to affect and cause variations in the walkability for the region. The configuration of some living facilities has a low correlation with walking distance, although our research results show that schools and hospital living facilities were found to have weighted scores of 85.00 or higher, while schools and hospitals are essential needs of life. In China, the government regulates the schooling of urban children according to their place of residence. Schools are divided into districts, which are less affected by walking distance. In addition, the imperfect medical system in China’s communities makes residents more trusting of the top three hospitals. Residents’ demand for hospitals has little to do with walking distance. Therefore, future research can consider reducing the weight coefficients for schools and hospitals.

In addition, the weight scores for catering, retail and department stores, and public service living facilities were around 88.00–95.00. The advantage of having more of these facilities is that it provides the residents with more options. The built environment of Zhongshan Road is complete, and there is no shortage of these types of facilities. 

The results also show that personal leisure facilities have a considerable impact on the walking index. The average score of bookstores is 74.00, and the average score of parks is 69.00, indicating that the parks and bookstores in the study area are inadequately allocated, and there is an imbalance in regional allocation. The built environment quality and construction density of each area may also have specific effects. The lack of recreational and leisure facilities indicates that, in addition to life services, recreational and spiritual service facilities are highly important for urban residents. Modern urban planning and design should give more attention to the recreational and spiritual needs to satisfy the growing and diversifying needs and demands of residents.

### 4.3. Optimization of Street Walk Score Algorithm

After obtaining the general walking index, a correction factor is needed to attenuate the index (including the newly added green vision index this time). The attenuation coefficient for intersection density is 3–5%. The density of existing intersections does not effectively promote walking. Infrastructure improvements to increase road network density would have to be undertaken to promote walking behavior. The attenuation coefficient for block length is 1–3%. Pedestrians and street spaces are closely connected, creating an easy form of walking travel. The attenuation coefficient for green vision rate is 3–5%. The average green viewing rate is 20.48%, indicating a low level of street greening.

Comparing the changes in the walking index before and after adding the green viewing rate index, there was a significant shift in the high-scoring area in Zhongshan Road. The walking scores around the western railway station declined significantly, indicating the need to include a green viewing rate indicator. Street greening has a certain practical significance for the quality of the walking environment. Streets with higher scores can provide examples for pedestrian system planning. Street-level greening has a certain reference significance for the path selection of individuals for outdoor activities and physical exercise. Other parameters affecting walkability can be integrated into the walk score algorithm, such as natural environmental factors (e.g., topography, water bodies), living group factors (e.g., resident age, occupation, and living density), and road traffic factors (e.g., vehicle flow, traffic pollution, public transportation conditions). 

### 4.4. Limitations of the Study

Several limitations of the study should be noted. First, walking speed is not fully representative of the walking ability of all inhabitants of the region. The standard walking speed was used to simplify calculations, but different age groups may have significant variations in pacing and acceptable walking distance. This simplification in our analysis may have significant effects on the calculations. Second, schools and general hospital facilities are affected by policies and residents’ psychological needs in China, and their correlation with walking distance is low, and their weight coefficients can be appropriately reduced. Third, the green vision rate is greatly affected by the season (especially in northern China). In this study, the streetscape picture used was taken at the end of the summer. For autumn and winter, however, there is basically no landscape greenery, except for evergreen trees. Therefore, street greening needs to further define the scope and methods of research for walkability optimization. Our research aims to provide residents with a comfortable walking environment and can be used to support planning methods for the construction of smart cities. However, our current research data can only provide suggestions for urban design at the block level. The architecture of urban systems at larger scales can be carried out in future research.

## 5. Conclusions

Our research evaluates street walkability using open-source POI data and street view images to analyze the rationality and accessibility of facility configuration for residents in Zhongshan Road. The main conclusions of the study are as follows:

The average walking index of Zhongshan Road is 79.74, showing a high trend in the west and a low trend in the east, and a high trend in the south and a low trend in the north. Using the residential area as a starting point, the living facilities in the area can be reached on foot within 15 min. The street’s current configuration comprises small blocks, which is favorable for later urban design updates. 

According to the general walking index, western stations and southern coastal areas have higher scores, and living facilities are well equipped; old northern and eastern residential areas have lower scores. Among them, the average weight of bookstores is 0.74, and the average weight of parks is 0.69. The quantity and configuration cannot meet the needs of residents’ daily leisure activities, and the accessibility of these two types of facilities is not convenient for residents’ daily life . It is necessary to appropriately supplement bookstores or similar facilities in community parks to improve daily facilities and services.

The average green viewing rate of Zhongshan Road is 20.48%, which is lower than the best visual perception value of 25.00%. The walking index was 79.74 before adding the green vision index, and 76.73 after the green vision index inclusion. The high-scoring area shifted from the west to the south, and the walking index in the west had the most significant decline. The inclusion of the green vision index allows for the evaluation of the accessibility of facilities and the friendliness of the physical space supporting walking. The metric can also be used to reflect the environmental quality of the walking activity space and objectively quantify the comfort level of people’s perception of street green spaces.

## Figures and Tables

**Table 1:** Overview of Zhongshan Road, Shinan District, Qingdao.

**Administered community**	**Population Size**	**Related Content**
	Over 5500	Guanhaishan Community, Pingdu Road Community, Zhejiang Road Community, Zhongshan Road Community, Hubei Road Community
	Below 5500	Shanxian Road Community, Tianjin Road Community, Henan Road Community, Guangxi Road Community, Jinan Road Community
Business economy	The commerce and trade economy is developed, with commercial outlets on both sides of the road, and “time-honored brands” such as Chunhelou, Hongrentang, and Shengxifu are located in the jurisdiction.	
Cultural tourism	There are many tourist attractions, such as the Zhanqiao, the Sixth Bathing Beach, the Catholic Church, Lao She Park, Guanhai Mountain, and other European-style buildings such as Wang Tongzhao, the former residence of Confucius, and the Jiaoao Governor’s Mansion. It is close to the railway station and has convenient transportation.	
Street culture	Chess competitions held on holidays, Zhongshan chessboard, Qingdao Summer Art Festival, International Beer Festival, Shinan Spring Art Show, and others.	
Ethnic fusion	The Han nationality in Shinan District accounts for 99.25% of the total population, and there are 24 ethnic minorities, including Manchu, Mongolian, Hui, Tibetan, Uygur, and Xibe.	
Climate overview	Shinan District is located in the northern temperate monsoon region, with a temperate monsoon climate, with humid air, abundant rainfall, and moderate temperature.	

**Table 2:** Improved facility classification and weight table.

Facility Classification		Classification Weight	Weight
Education	School	1.5	1.5
Health care	Hospital	1	1.5
	Pharmacy	0.5	
Repast	Restaurant	1.75	3.5
	Bakery/coffee shop/tea house	1.75	
Retail department store	Supermarket/mall	1.5	3
	Convenience store	1.5	
Personal leisure	Bookstore	1	3.5
	Park	1	
	Sports/entertainment	1.5	
Public service	Bank/ATM	0.75	2
	Post office	0.25	
	Barbershop	1	
Total	15		

Note: Libraries belong to the bookstore category, and theaters, cinemas, etc., belong to the category of sports/entertainment.

**Table 3:** Green vision rate.

Description of Greening Degree	Green Vision Rate (%)
Not green	≤20
Normal green	(20–40)
Green	(40–50)
Very green	>50

Note: When greenery reaches 25% in the human field of vision, people feel the most comfortable. When the green vision rate is greater than 0.25, the location is deemed a comfortable green street.

**Table 4:** Comparison table of intersection density, block length, and green view rate attenuation rate.

Intersection Density (Pcs/km^2^)	Attenuation Rate (%)	Block Length (m)	Attenuation Rate (%)	Green Vision Rate (%)	Attenuation Rate (%)
>77	0	<120	0	25 (Optimal)	0
58–77	1	120–150	1	>25 (Comfortable street)	1
47–58	2	150–165	2		
37–47	3	165–180	3	(20,25) (Normal green)	3
23–35	4	180–195	4		
<23	5	>195	5	<20 (Not green)	5

**Table 5:** Walk score evaluation grade.

Walk Score	Description	
90–100	Walkers’ paradise	Daily travel can be carried out by walking
70–89	Very walkable	Most facilities can be reached on foot
50–69	Average walkability	Some facilities are within walking distance
25–49	Poor walkability	Fewer facilities within walking range
0–24	Car dependence	Almost all trips rely on cars

**Table 6:** Weight statistics of living facilities in low-score communities.

Facilities	72 HDY	YHBL	LNLY	LBXTD	PHL	WML	AKL	HXL	JSL	PKWL	SJL	YCSZ	JTLG	GHLBS	YSLXQ
School	0.825	0.825	1.32	1.32	1.32	1.32	1.32	1.32	1.32	1.32	1.32	1.32	0.825	0.825	1.32
Hospital	0.88	0.88	0.88	0.88	0.88	0.88	0.88	0.88	0.88	0.88	0.88	0.88	0.88	0.88	0.55
Pharmacy	0.5	0.5	0.5	0.5	0.5	0.5	0.5	0.5	0.5	0.5	0.5	0.5	0.44	0.5	0.5
Restaurant	1.75	1.75	1.75	1.75	1.75	1.75	1.75	1.75	1.75	1.75	1.75	1.75	1.75	1.75	1.75
Bakery/coffee shop/tea house	1.75	1.75	1.75	1.75	1.75	1.75	1.75	1.75	1.75	1.75	1.75	1.75	1.75	1.75	1.75
Supermarket/mall	1.5	1.5	1.5	1.5	1.5	1.5	1.5	1.5	1.5	1.5	1.5	1.5	1.32	1.5	1.5
Convenience store	1.5	1.5	1.5	1.5	1.5	1.5	1.5	1.5	1.5	1.5	1.5	1.5	1.5	1.5	1.5
Bookstore	0.84	0.84	0.84	0.84	0.52	0.84	0.52	0.87	0.52	0.52	0.52	0.52	0.84	1	1
Park	0.55	0.55	0.88	0.88	0.55	0.55	0.55	0.88	0.88	0.55	0.55	0.55	0.55	1	0.88
Sports/entertainment	1.5	0.55	1.5	1.5	1.32	1.32	1.32	1.32	1.32	1.32	1.32	1.32	1.32	1.5	1.5
Bank	0.75	0.75	0.75	0.75	0.66	0.66	0.66	0.66	0.66	0.66	0.66	0.66	0.41	0.75	0.75
Post office	0.22	0.22	0.22	0.22	0.13	0.13	0.13	0.13	0.13	0.13	0.13	0.13	0.13	0.25	0.25
Barbershop	1	1	1	1	1	1	1	1	1	1	1	1	1	1	0.88

Note: 72 HDY—Compound 72; YHBL—Yuhou Beili; LNLY—Luneng Residence; LBXTD—Lubang Xintiandi; PHL—Pingheli; WML—Wenmingli; AKL—Ankangli; HXL—Hexingli; JSL—Jishanli; PKWL—Pingkangwuli; SJL—Sanjiangli; YCSZ—Yucheng Villa; JTLG—Family Inn; GHLBS—Guanhai Old Villa; YSLXQ—Yishui Road Community.
